# The relationship between perseverative thinking, proactive control, and inhibition in psychological distress: a study in a women’s cohort

**DOI:** 10.1038/s41598-023-46713-9

**Published:** 2023-11-07

**Authors:** Lorenzo Mattioni, Marcantonio M. Spada, Francesca Ferri, Carlo Sestieri

**Affiliations:** 1 Department of Neuroscience, Imaging and Clinical Sciences - and ITAB, Institute for Advanced Biomedical Technologies, University G. d’Annunzio di Chieti-Pescara, Via Dei Vestini 11, 66100 Chieti, Italy; 2 School of Applied Sciences, London South Bank University, London, UK

**Keywords:** Psychology, Emotion, Stress and resilience

## Abstract

Cognitive control is a core feature of several mental disorders. A recent account poses that health problems may derive from proactive forms of cognitive control that maintain stress representation over time. The working hypothesis of the present study is that psychological distress is caused by the tendency to select a particular maladaptive self-regulation strategy over time, namely perseverative thinking, rather than by transient stimulus–response patterns. To test this hypothesis, we asked 84 women to carry out a battery of standardized questionnaires regarding their tendency to undertake perseverative thinking and their level of psychological distress, followed by cognitive tasks measuring the tendency to use proactive versus reactive control modality and disinhibition. Through a series of mediation analyses, we demonstrate that the tendency to use proactive control correlates with psychological distress and that this relation is mediated by perseverative thinking. Moreover, we show that the relation between low inhibitory control and psychological stress is more strongly mediated by perseverative thinking than impulsiveness, a classical construct that focuses on more transient reactions to stimuli. The present results underline the importance of considering psychological distress as the consequence of a maladaptive way of applying control over time, rather than the result of a general deficit in cognitive control abilities.

## Introduction

In real-world situations, the ability to predict future events provides a great evolutionary advantage. Humans possess cognitive mechanisms that allow them to vividly represent past experiences and further manipulate these representations to foresee, plan, and shape future events^1^. Similarly, adaptively anticipating emotions that will be associated with an upcoming stressful event helps individuals deal with the actual stressor^2,3^.

However, engaging in making predictions has the potential to become maladaptive and breed psychological distress when it takes the form of “perseverative thinking”, i.e. the inflexible, repetitive, relatively uncontrollable thinking that focuses on emotion-relevant content^4^. This activity is a dysfunctional attempt to obtain information about oneself and the present context and results in the emergence of cognitive biases that amplify and maintain distress-related representations over time^5^. Perseverative thinking can be seen as attempts to engage in emotion regulation, i.e. the activation of a goal to influence the emotion trajectory in both explicit and implicit manner^6^. It involves the identification of emotional distress and the selection and implementation of an emotion regulation strategy but also the evaluation of this strategy in order to maintain, change, or stop it^7^.

The tendency to engage in perseverative thinking is explained by more or less implicit metacognitive beliefs that it is both a useful strategy to regulate negative affect and an inevitable consequence of distress^8,9^. For example, an individual may choose to worry or ruminate over other strategies to cope with distress. Worry is defined as the predominance of negatively valenced verbal thought activity reflecting an attempt at abstract problem-solving concerning the potentially negative outcomes of a situation^10^. Rumination is instead defined as a passive and past-oriented focusing on one’s symptoms, their causes, and consequences as an attempt to gain insight^11^. Different kinds of perseverative thinking may be oriented toward the past or the future and may involve different disorder-specific peculiarities, but they all depend on the same neural mechanisms that allow event recollection, imagination, and prediction^12,13^.

It has been proposed that perseverative thinking represents a behavioural hallmark of inefficient allostasis^14^, which refers to the process of distress adaptation^15^ and represents an adaptive autonomic anticipation of potentially stressful events^16^. In particular, perseverative thinking has been found to mediate prolonged physiological responses to stressors^4,17^ by affecting the hemodynamic profile^18,19^, the immune function^20,21^, and the endocrine system^22,23^.

For example, the worry about a distant situation triggers the activation of allostatic processes that prime the body as if the distressing event was imminent. The duration of worry plays a mediating role in the impact of stressors^24^. The diminished ability to inhibit negative representations, along with the associated physiological response, may thus lead to a maladaptively prolonged stress response. According to this view, the primary pathogenic pathway is not the transient exaggerated physiological response to specific stressors but rather the total amount of distress-induced physiological activation over time^25^. Importantly, while stress promotes adaptation in the short run, a failed shutoff of mediators after a stressful situation eventually leads to “wear and tear” on the body^26^. For its involvement in a wide range of disorders, perseverative thinking is now considered a transdiagnostic feature^27^ that reflects a maladaptive interaction of self-referential and affective processing with cognitive control and autonomic arousal^28^.

### Proactive control and psychological distress

The success in regulating the distress response is determined by the correct implementation of cognitive control^29^. This type of self-regulation requires the integration of basic aspects of executive function, such as working memory operations, behavioural inhibition, and task-switching^30^. Adaptive behaviour emerges from a trade-off between cognitive control and flexibility. On the one hand, without the ability to protect goals from interfering stimuli and prepotent responses, an organism would suffer from distractibility. On the other hand, without the ability to flexibly reconfigure cognitive sets and response dispositions, the individual would be unable to adapt to changing circumstances and would suffer from perseveration and behavioural rigidity^31^. Cognitive control operates via two distinct and independent modes^32,33^, namely reactive and proactive control. In the first mode, attention is recruited as a late correction mechanism that is mobilized only as needed in a just-in-time manner, such as when a high interference event is detected. Such a transient activity might reflect the bottom-up reactivation of task goals. The second mode is a form of goal-driven selection, in which relevant information is actively maintained in a sustained manner to optimally bias attention, perception, and action systems before the occurrence of upcoming events. Under proactive control, goal representation is triggered in advance of its implementation and maintained continuously during periods in which it is required, optimizing preparation while minimizing distractions. In this respect, it is possible to consider perseverative thinking as prolonged implementation of proactive control in a maladaptive attempt to regulate emotions^34^.

Previous studies have investigated the relationship between psychological distress and *deficits* of proactive control but found no evidence that behavioural deficits in proactive control explain depression^35^, anxiety^36^, or addiction^37^. A possible reason for this null result is that the influence of proactive control over psychological distress crucially depends on expectations about when to use it^38^. As a matter of fact, different expectations can shape subsequent regulatory responses in adaptive or maladaptive ways. For example, increasing positive expectancies can adaptively fuel anticipatory processes, leading to optimal levels of proactive control regarding a stressful event. In contrast, decreasing expectations concerning the ability to cope with the stressor before its actual detection likely influences its anticipation, leading to the activation of maladaptive schemas and self-reflective negative thoughts^29^. In our view, a negative outcome derives from a *misuse,* rather than a deficit, of proactive control, which becomes maladaptive when taking the form of perseverative thinking. Consequently, the tendency to adopt a proactive modality is not maladaptive per se, but only when associated with sustained distress representation.

### Inhibitory control and stress response

Traditionally, studies have focused on the role of reactive control in explaining psychological distress. Reactive control is less specific than proactive control and involves the global inhibition of prepotent responses. It’s usually measured in tasks, like the Go/No-Go, that require the unexpected request to suppress a movement plan, thereby emphasizing motor inhibition. In particular, impulsiveness, the tendency to engage in rapid, unplanned reactions to internal or external stimuli, without regard to the negative consequences, is generally considered a key link between diminished inhibitory control and psychopathology^39,40^. According to this view, deficits in the ability to inhibit prepotent responses reflect the tendency to engage in impulsive behaviours^41,42^, eventually leading to psychological distress^43^.

However, the link between inhibitory control and psychological distress may be more complex than previously hypothesized. Firstly, inhibitory control is not a unitary construct. It involves the ability to control one’s behaviour, but also one’s attention, thoughts, and/or emotions to override a strong internal predisposition, or external lure, and do what’s more appropriate^44^. Indeed, tasks tapping on inhibition-related functions involve multiple cognitive processes^45^, with differences in performance largely explained by working memory capacity^46^. Also at the neural level, the brain areas responding during inhibition tasks appear more generally associated with working memory capacity rather than being specifically associated with inhibitory control^47^. Secondly, a view that focuses on how individuals transiently respond to stimuli and impulses may not fully capture the dynamic aspects of control^48,49^ that contributes to long-term distress^17^.

An alternative view poses instead that psychopathological symptoms are better conceptualized by adopting a sustained perspective^50^. Accordingly, an unspecific inhibition only occasionally pertains to impulse control, while effective real-world control depends on a range of interconnected cognitive abilities^51^. Within this framework, we propose that perseverative thinking represents the connection between diminished inhibitory control and psychological distress, as it involves sustained engagement in distress-related information processing^52^. In other words, a low level of inhibitory control is not maladaptive per se but only when associated with a tendency for perseverative thinking. This hypothesis is supported by the link, observed at both the behavioural^53,54^ and neural^55^ level, between low inhibitory control and the inability to deactivate previous thoughts, which is a crucial feature of perseverative thinking. Furthermore, inhibitory control also involves the inhibition of allostatic processes^4^ that are fundamental for perseverative thinking^56^. Also in this case overlapping areas are thought to perform similar computations whether to inhibit an operant or an emotional response^57^.

### Working hypotheses

The present study focuses on the effect of the interaction between cognitive control and perseverative thinking in the development and maintenance of psychological distress. Specifically, we hypothesize that an increased use of proactive control becomes maladaptive when coupled with perseverative thinking (Hypothesis 1). Consequently, we anticipate that perseverative thinking acts as a complete mediator in the relationship between proactive control and psychological distress. This suggests that individuals who lean towards using the proactive control modality may report higher levels of psychological distress only when this tendency leads to increased perseverative thinking. The innovation of this hypothesis consists in the operationalization of proactive control in terms of the *tendency* to use this modality, rather than in terms of the *performance* in a cognitive control task. We further predict that perseverative thinking serves as a complete mediator in the relationship between decreased inhibitory control and psychological distress, above and beyond the role of impulsiveness (Hypothesis 2). This hypothesis stems from the consideration that low inhibitory control becomes maladaptive when contributing to maintaining distress over time more than when simply increasing transient reactions to stimuli and impulses. The innovative feature of this second hypothesis is to consider low inhibitory control not as problematic per se, but only when involving further cognitive processes that perpetuate mental suffering.

To test our hypotheses, we recruited 84 volunteers from a university student population and asked them to complete online questionnaires pertaining to various aspects of perseverative thinking, psychological distress, and impulsiveness. Additionally, participants performed online cognitive tasks designed to assess their use of reactive vs. proactive cognitive control modes, as well as their inhibitory control. This approach allowed us to conduct a series of mediation analyses, assessing the impact of perseverative thinking on the relationship between cognitive control and psychological distress. Research has indicated that women, compared to men, exhibit a higher inclination toward proactive processing^58^, demonstrate quicker reaction times in tasks involving behavioural inhibition^59^, and display a heightened propensity for engaging in perseverative thinking^60,61^. In light of this, women are likely more exposed to factors related to the primary study variables. Therefore, we recruited a sample consisting solely of female participants to exclude the influence of gender differences on the data.

## Results

### Hypothesis 1

As a prerequisite for running the mediation models, we verified the presence of a significant correlation between the measures included in the analysis: perseverative thinking (PTQ), cognitive attentional syndrome (CAS-1), psychological distress (DASS-21), emotion dysregulation (DERS), and proactive control (PBI). The correlation coefficients are reported in Table 1.

**Table 1:** Bootstrapped Pearson correlations between measures of perseverative thinking and related clinical features, psychological distress, emotion dysregulation, and proactive control.

	1	2	3	4	5
1—Perseverative thinking (CAS-1)	–				
Upper bound	–				
Lower bound	–				
2—Perseverative thinking (PTQ)	0.64^a^	–			
Upper bound	0.75	–			
Lower bound	0.49	–			
3—Emotion dysregulation (DERS)	0.64^a^	0.76^a^	–		
Upper bound	0.75	0.85	–		
Lower bound	0.49	0.65	–		
4—Negative affect (DASS-21)	0.55^a^	0.63^a^	0.77^a^	–	
Upper bound	0.68	0.74	0.86	–	
Lower bound	0.40	0.49	0.65	–	
5—Proactive control (PBI for reaction times)	0.25^a^	0.24^a^	0.24^a^	0.22^a^	–
Upper bound	0.40	0.44	0.43	0.39	–
Lower bound	0.10	0.04	0.06	0.05	–

^a^Bootstrapped confidence intervals not including zero.

**Figure 1:**
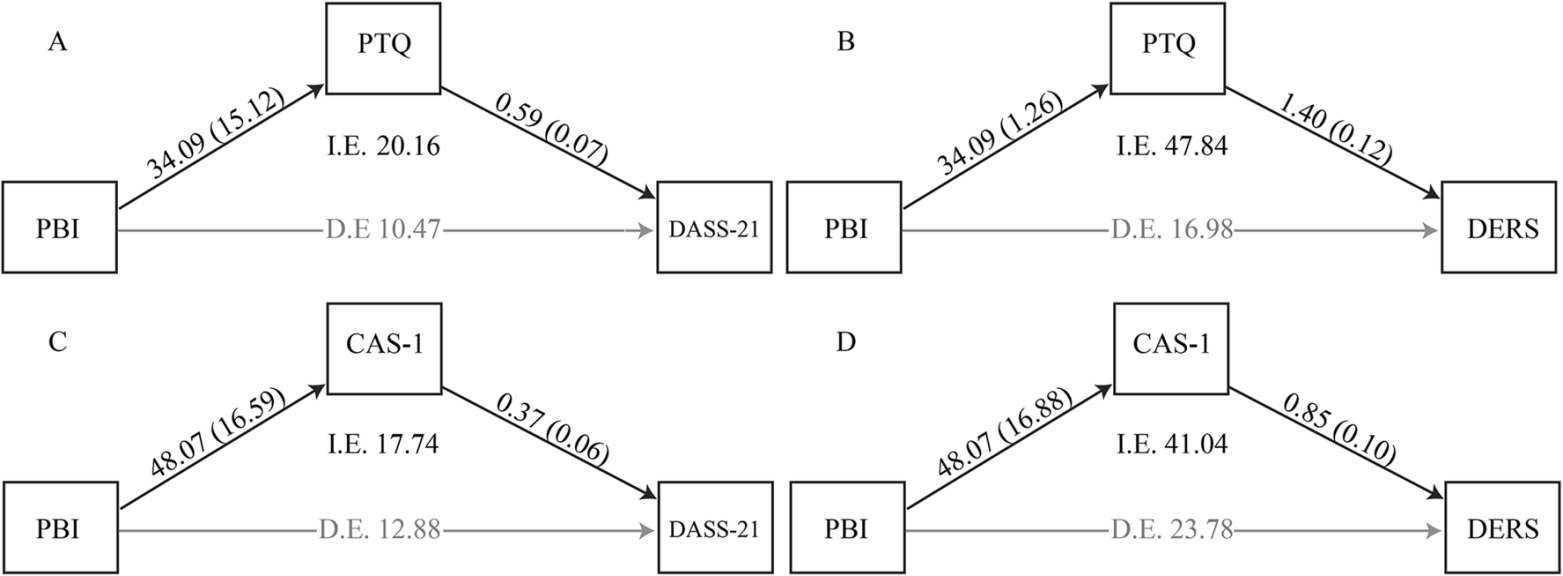
Mediation models of PBI as a predictor of DASS-21 and DERS through PTQ and CAS-1. *I.E.* indirect effect, *D.E.* direct effect, black arrows indicate that the effect is significant; grey arrows indicate that the effect is not significant; the numbers near the arrows indicates standardized parameter estimates (standard errors in parentheses).

The results of 95% bootstrapped CI to further assess the significance of direct and indirect effects are shown in Table 2. In line with our hypothesis, the first four mediation analyses confirmed the role of perseverative thinking as a significant mediator of the association between proactive control and psychological distress. In particular, the model using perseverative thinking (PTQ) as a mediator of the association between proactive control mode (PBI) and psychological distress (DASS-21 and DERS) (Fig. 1A,B) indicated that the direct association was not significant regardless of the measure of psychological distress used. Instead, the indirect effect mediated by perseverative thinking (PTQ) was significant for both measures of psychological distress (Fig. 1A,B). The same pattern was observed when considering CAS-1 as a measure of perseverative thinking (Fig. 1C,D). Also in this case, the direct association between proactive control mode (PBI) and psychological distress (CAS-1) was not significant, regardless of the measure of psychological distress (DASS-21 and DERS). Again, the indirect effect mediated by CAS-1 was significant for both the DASS-21 and the DERS. To summarize, the present data support a model according to which proactive control causes perseverative thinking, which, in turn, causes negative affect and emotion dysregulation. Importantly, the control mediation analyses testing for an inverse relationship between psychological distress and proactive control mode indicated the absence of any significant effect, as shown in Table 3.

**Table 2:** Direct and indirect effects of the mediation analyses.

Paths	Effect	SE	95% bootstrapped CI	
			Lower bound	Upper bound
Proactive control → perseverative thinking → negative affect	20.16^a^	14.88	1.03	60.22
Proactive control → negative affect	10.47	2.31	− 15.34	31.30
Proactive control → perseverative thinking → emotion dysregulation	47.84^a^	22.19	8.62	96.22
Proactive control → emotion dysregulation	16.98	19.88	− 25.09	53.59
Proactive control → CAS → negative affect	17.74^a^	6.94	6.26	33.49
Proactive control → negative affect	12.88	11.57	− 10.53	35.16
Proactive control → CAS → emotion dysregulation	41.04^a^	15.05	14.07	72.91
Proactive control → emotion dysregulation	23.60	22.68	− 20.16	70.07

^a^Bootstrapped confidence intervals not including zero.

**Table 3:** Direct and indirect effects of the control mediation analyses.

Paths	Effect	SE	95% bootstrapped CI	
			Lower bound	Upper bound
Negative affect → perseverative thinking → proactive control	8E−4	7E−4	− 7E−4	2E−3
Negative affect → proactive control	8E−4	1E−3	− 1E−3	3E−3
Emotion dysregulation → perseverative thinking → proactive control	4E−4	5E−4	− 6E−4	1E−3
Emotion dysregulation → proactive control	5E−4	6E−4	− 2E−3	3E−3
Negative affect → CAS → proactive control	9E−4	4E−4	− 1E−4	3E−3
Negative affect → proactive control	9E−4	8E−4	− 7E−4	3E−3
Emotion dysregulation → CAS → proactive control	4E−4	3E−4	− 2E−4	9E−4
Emotion dysregulation → proactive control	5E−4	6E−4	− 4E−4	2E−3

### Hypothesis 2

The correlation coefficients for all the correlation analyses between measures of perseverative thinking (PTQ), psychological distress (DASS-21) impulsiveness (BIS), and inhibitory control (total error in No-Go Trials) are reported in Table 4.

**Table 4:** Bootstrapped Pearson correlations between measures of perseverative thinking, psychological distress, and disinhibition.

	1	2	3	4
1—Perseverative thinking (PTQ)	–			
Upper bound	–			
Lower bound	–			
2—Negative affect (DASS-21)	0.66^a^	–		
Upper bound	0.77	–		
Lower bound	0.52	–		
3—Impulsiveness (BIS-11)	0.33^a^	0.27^a^	–	
Upper bound	0.53	0.46	–	
Lower bound	0.12	0.07	–	
4—Disinhibition (total error in no go trials)	0.26^a^	0.28^a^	0.30^a^	–
Upper bound	0.46	0.46	0.45	–
Lower bound	0.06	0.10	0.17	–

^a^Bootstrapped confidence intervals not including zero.

**Figure 2:**
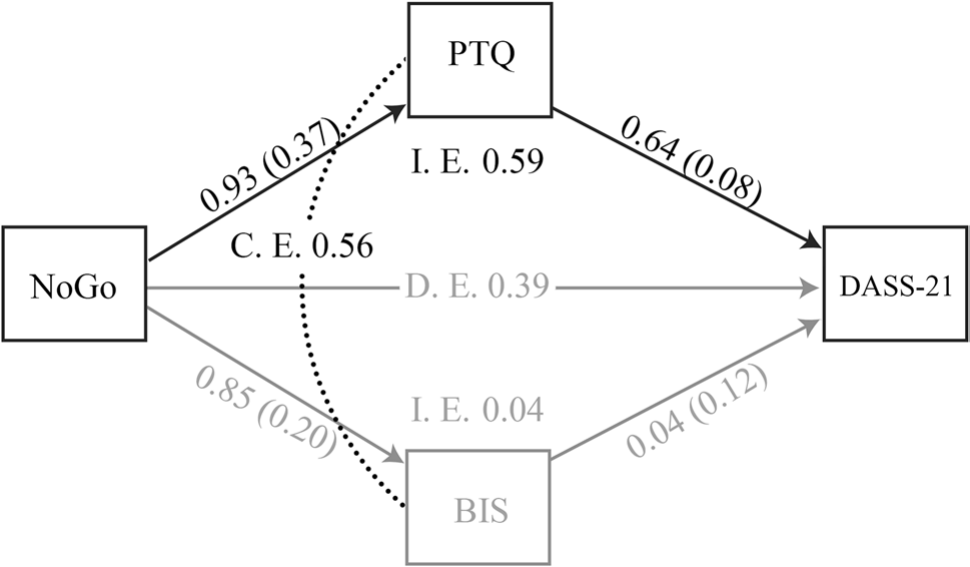
Mediation model of No Go as a predictor of DASS-21 through PTQ. *I.E.* indirect effect, *D.E.* direct effect, *C.E.* contrast effect, which is PTQ effect minus BIS effect; black arrows indicate that the effect is significant; grey arrows indicate that the effect is not significant; dotted line indicate the contrast between the mediators; the numbers near the arrows indicates standardized parameter estimates (standard errors in parentheses).

The results of the bootstrapped 95% confidence interval to assess the significance of direct and indirect effects are shown in Table 5. As hypothesized, the parallel mediation of PTQ and BIS on the effect of No Go errors on DASS-21 results was significant, whereas the direct effect did not reach the significance level. Furthermore, when considering the indirect effects of PTQ and BIS separately, a significant effect was only observed for the PTQ. The bootstrap pairwise comparison analysis confirmed that the specific indirect contrast effect of PTQ and BIS was significant, meaning that PTQ mediates the effect of No-Go errors on DASS-21 significantly better than BIS (Fig. 2). As the construct of impulsiveness has been previously divided into attentional, motor, and non-planning components^62^, we conducted further mediation analyses considering each component in isolation. Importantly, PTQ remained a significant mediator while attentional, motor, and non-planning impulsiveness all resulted in non-significant mediating effects. The bootstrap pairwise comparison analyses resulted in a significantly greater effect of PTQ in mediating this relationship than both motor and non-planning impulsiveness. The comparison was not significant for attentional impulsiveness, possibly because this subscale may underly general personality traits associated with impulsiveness rather than an independent factor per se, resulting in a less “pure” construct^62^.

**Table 5:** Direct and indirect effects of the mediation analyses.

Paths	Effect	SE	95% Bootstrapped CI	
			Lower bound	Upper bound
Disinhibition → negative affect	0.39	0.32	− 0.25	1.03
Disinhibition → perseverative thinking → negative affect	0.59^a^	0.24	0.12	1.07
Disinhibition → impulsiveness → negative affect	0.04	0.11	− 0.19	0.23
Perseverative thinking – impulsiveness	0.56^a^	0.26	0.04	1.09
Disinhibition → negative affect	0.42	0.27	− 0.07	1.00
Disinhibition → perseverative thinking → negative affect	0.55^a^	0.23	0.11	1.00
Disinhibition → attentional impulsiveness → negative affect	0.14	0.11	− 0.02	0.43
Disinhibition → motor impulsiveness → negative affect	− 0.08	0.11	− 0.35	0.10
Disinhibition → non-planning impulsiveness → negative affect	− 0.02	0.10	− 0.18	0.15
Perseverative thinking—attentional impulsiveness	0.41	0.28	− 0.18	0.92
Perseverative thinking—motor impulsiveness	0.63^a^	0.24	0.18	1.14
Perseverative thinking—non-planning impulsiveness	0.57^a^	0.24	0.09	1.07

^a^Bootstrapped confidence intervals not including zero.

To summarize, the analyses confirmed that a lack of inhibitory control is associated with perseverative thinking, which in turn is associated with psychological distress, whereas impulsiveness does not mediate the relationship between inhibitory control and psychological distress.

## Discussion

### The relation between proactive control, perseverative thinking, and psychological distress

Humans excel in the ability to anticipate potential threats and strategically allocate resources to address them. However, anticipation comes at a cost. It has been suggested that repetitive and uncontrollable thinking that focuses on emotion-relevant content can disrupt anticipatory self-regulation^63^. This maladaptive activation of proactive control is characterized by unproductive processing of information, specifically fixating on emotionally-valenced thoughts while maintaining a low-construal perspective^34^. In this context, we consider perseverative thinking as the misapplication of proactive control mechanisms to cope with distress-related thoughts and situations.

The present findings suggest that the disposition towards perseverative thinking serves as a complete mediator in the relationship between the use of a proactive control mode and psychological distress. Importantly, this effect is monodirectional, with proactive control leading to perseverative thinking, which in turn leads to mental suffering (and not vice versa). Our data support the hypothesis that perseverative thinking plays a causal role in transmitting the effect of proactive control toward psychological distress. At the same time, the results imply that a deficit in proactive control does not necessarily result in distress^38^, but only when activating cognitive processes that maintain emotionally-salient representations over time.

Before an arousing event, an individual’s expectations regarding their regulatory abilities can be either adaptive or maladaptive^29^. Efficient regulation requires anticipating needs and preparing to satisfy them before they arise^64^. Aspinwall and Taylor^65^ proposed a framework for proactive coping consisting of five phases: i. development of anticipatory schema, ii. search internal and external inputs for distress-related cues, iii. detection and appraisal of the stressor, iv. development of a coping plan, and v. analysis of plan efficacy. Proactive control always implies the anticipatory use of resources to pursue a specific goal, sacrificing efficiency for efficacy^66^. In this view, perseverative thinking may cause a waste of resources in each phase of proactive coping, in terms of: i. excessive cognitive and allostatic activity to prepare for long-term goals or distant threats^67^, ii. constant and biased monitoring of stressors^8^, iii. overrepresentation of salient information^56^, iv. difficulty of elaborating constructive plans to deal with stressors^34^, and v. ineffective analysis of plan efficacy due to biased memory updating towards prior schemas^68,69^.

These effects can be generalized through the allostatic load model^25^, which emphasizes that the primary index of pathology is not the difference between the baseline activity and a specific physiological activation, but rather the total amount of distress-induced physiological activation over time^70^. In real life, environmental stressors are transient and occur only occasionally. However, perseverative thinking can prolong the stress response by continuously elevating the organism’s perceived need for regulation due to the persistent representation of salient information^4^.

### The relation between inhibitory control and perseverative thinking

In our proposed framework, cognitive control becomes maladaptive when maintaining stress-related information. Consequently, we have demonstrated that a tendency to use a proactive control mode is associated with a propensity for repetitive thinking, ultimately resulting in elevated levels of psychological distress. A second hypothesis stemming from our framework poses a similar role of perseverative thinking in explaining the relationship between psychological distress and a less specific control modality, i.e. inhibitory control as measured in the Go/No-Go task. The present results are entirely consistent with this prediction.

Motor inhibition is closely related to the inhibition of memories^55^ and emotional responses^57^, both of which are fundamental aspects of perseverative thinking^56,71^. Thus, continuous attempts to inhibit emotionally salient thoughts, internal emotional states, and both physiological and behavioural responses may lead to distress by constantly requiring cognitive and physiological work^72^. Indeed, response inhibition in the face of uncertainty is considered a precursor of negative coping, which perpetuates perseverative thinking^73^. Therefore, lower inhibitory control may imply a greater inclination toward redundant stressor representation. For instance, both worry^74^ and rumination^75^ are associated with low inhibitory control. Additionally, inhibitory control abilities play an indispensable role in cognitive flexibility^76,77^. This concept refers to the readiness with which one can selectively switch between mental processes to generate appropriate responses^78^. Cognitive flexibility is a general property of a cognitive system that emerges from a complex interplay between cognitive and sensorimotor mechanisms^79^ and is inversely related to perseverative thinking, which is characterized by rigid cognitive patterns^80^.

The presented conceptualization of the maladaptive use of cognitive control offers greater explanatory power compared to the classical framework that focuses on how impairments in motor inhibitory control lead to impulsiveness^41^, which in turn leads to higher levels of psychological distress^43^. This conventional perspective considers the maladaptive components of reduced inhibition as a consequence of dysfunctional impulse control. Instead, we posit that this straightforward viewpoint fails to account for important dynamic features of sustained distress. Accordingly, we also predicted that such mediation would not be affected by impulsiveness scores. As a matter of fact, our findings revealed that impulsiveness is not a significant mediator for the effect of inhibitory control on psychological distress and that perseverative thinking significantly outperformed impulsiveness in explaining this relationship. Thus, our results not only confirm that perseverative thinking fully mediates the negative relation between inhibitory control and psychological distress but also establish that this negative effect does not result from an increase in impulsivity, This was true regardless of considering impulsiveness as a general measure or separately considering its subcomponents^62^.

Therefore, the notion that reduced inhibitory control makes individuals more impulsive and, consequently, more distressed may only represent a part of the story. Indeed, a paradigm in which participants stop a response outright when signaled to do so is the most direct way to investigate control mechanisms and serves as a valuable starting point for mapping the neural architecture of cognitive control. Nevertheless, this global reactive stopping is only occasionally relevant in real-world problems^51^. Hence, the maladaptive components of motor inhibition may not reflect a predisposition for a rapid and premature action without appropriate foresight, but a more general dysfunctional mechanism that implies sustained distress representation.

### Implications

The present findings suggest that everyday psychological distress may stem from the maladaptive use of cognitive control, specifically, perseverative thinking. In this respect, the study implies that proactive cognitive control and inhibition may be important targets for the treatment of psychological distress in clinical settings. In particular, emphasis could be placed on providing proactive and inhibitory skills related to flexibility (e.g., teaching when and how to use proactive control or instructing to voluntarily switch the attention away from repetitive thoughts), variability (e.g., training aimed at expanding the range of mental contents), and efficacy evaluation (e.g., instructing individuals on how to evaluate the reliability of a particular cognitive control activity). This approach would move beyond merely enhancing proactivity and general inhibitory capabilities. Within the context of psychological distress, the data imply that both proactive control and inhibition contribute to the inclination toward perseverative thinking, which can manifest, for example, as worry or rumination, depending on the specific focus. Future research may consider psychological distress as an outcome of various cognitive processes that share the common aspect of continuously selecting and implementing a particular proactive coping strategy, which redundantly involves stress-related representations.

Finally, our objective is to provide support to a global framework that accounts for how basic processes of cognitive regulation may be involved in the development and maintenance of psychological distress. The study harks back to a view of psychopathology that considers psychological distress as consisting of maladaptive cognitive and behavioural actions that are volitionally repeated during life, rather than a nosographic label to distinguish health from the disease based on how people react to particular stimuli.

### Limitations

One limitation of our study was the skewed distribution of DASS-21 scores, possibly due to the research being conducted in Italy between 20/10/2020 and 20/12/2021, during the COVID-19 global pandemic. The fast-changing dynamics of the situation may have influenced the participants’ state at the time of the experiment, a variable that we did not consider in our study. In addition, the selected Go/NoGo task, originally designed for adults with a history of cocaine use^81^, was found to be too easy for our volunteers, resulting in a ceiling effect in some participants. To address these issues, we employed bias-corrected bootstrap confidence intervals in our analyses, a method of hypothesis testing that minimizes bias related to non-normal sampling distributions^82^. Nonetheless, we believe that a more challenging Go/NoGo may yield even stronger results. Lastly, we opted to recruit only female participants to exclude gender influence in the data. Importantly, females may exhibit a greater susceptibility to the key variables under study than men. They tend to report higher levels of rumination^60^, indicative of a lower sense of control over emotions and significant life events and higher perceived responsibility for relationships^83^. Additionally, women report higher levels of worry than men^84^. Finally, female volunteers have reported increased concerns related to self-confidence, a more negative approach to problem-solving, and greater engagement in thought inhibition^61^, particularly among Italian university students^85^. In this context, women also seem to be more inclined than men to utilize a proactive control modality^33,58^. Thus, while we focused on a population expected to be particularly exposed to the central constructs of the present study, future research should replicate these results with male participants to account for potential gender differences.

## Conclusions

In this study, we have explored the relationship between executive control and psychological distress by examining the role of perseverative thinking. Our results demonstrate that the transmittance of change from proactive control to psychological distress occurs through perseverative thinking. Furthermore, we demonstrate that disinhibition is related to psychological distress, with perseverative thinking serving as a complete mediator, outperforming impulsiveness. Thus, the maladaptive aspects of motor inhibition may not solely result from a tendency toward actions lacking proper foresight but more generally stem from a dysfunctional mechanism that involves the persistence of distress-related representations. These findings suggest focusing on the mechanism that ends up selecting a particular pattern of proactive self-regulation strategies over time to explain mental suffering.

## Methods

### Participants

A total of 94 Italian native speakers (all women) between 18 and 30 years of age (mean age = 22.2; SD = 2.7) were sampled from a volunteer list of students willing to take part in behavioural experiments at the D’Annunzio University of Chieti, Italy. Informed consent was obtained from every participant. Eight participants did not complete the study, two were not able to perform the proactive control task while another two did not perform the inhibitory control task, leaving a final sample of 84 participants for each task. The sample size was estimated with the software MedPower^86^ for a desired power of 0.80, a medium effect size of 0.35 for every path of the mediation analysis, and an alpha of 0.05^87,88^. Participants received a €14 reimbursement for their participation in the study. The research project complies with the Declaration of Helsinki and was approved by the Ethics Committee of the Provinces of Chieti and Pescara on the 8th of October 2020 (verbal n. 22).

### Measures

Participants completed a battery of questionnaires, validated in Italian, on different aspects of perseverative thinking and psychological distress and two tasks aimed at measuring the tendency to use proactive control mode and impulsiveness. Means, standard errors, and score ranges are reported in Table 6.

**Table 6:** Means, standard errors, and score ranges of the variables used in the study.

	CAS-1	PTQ	DASS-21	DERS	BISS	PBI	No Go
M	59.68	29.49	23.25	76.77	57.57	0.01	3.07
se	1.85	1.35	1.32	2.57	1.07	0.01	0.38
Range	0–128	0–60	0–63	36–150	30–120	− 1 to 1	0–125

#### Cognitive attentional syndrome-1—CAS-1

The CAS-1^89,90^ is a 16-item self-report measure of perseverative thinking and underlying positive and negative metacognitive beliefs with satisfactory reliability and validity^91^. It measures four dimensions: worry/rumination, threat monitoring, coping behaviours, and metacognitive beliefs. The first two items reflect the amount of time spent worrying or “dwelling” on problems and focusing attention on threats. The next six items capture the frequency of unhelpful strategies used to cope with negative thoughts or feelings (e.g., “Tried not to think about things”), and the final eight items assess positive and negative metacognitive beliefs about the CAS (e.g., “Worrying helps me cope”; “I cannot control my thoughts”). Higher scores indicate greater levels of perseverative thinking.

#### Perseverative thinking questionnaire—PTQ

The PTQ^92^ is a 15-item self-report measure of perseverative thinking beyond any particular disorder-specific content with high internal consistencies and high re-test reliability^93^. The item pool comprises three items for each of the assumed process characteristics of perseverative thinking: repetitive (e.g., “The same thoughts keep going through my mind again and again”), intrusive (e.g., “Thoughts come to my mind without me wanting them to”), difficult to disengage from, unproductive (e.g., “I keep asking myself questions without finding an answer”), capturing mental capacity (e.g. “My thought prevent me from focusing on other things”). Higher scores indicate greater levels of perseverative thinking.

#### Depression anxiety and stress scale-21—DASS-21

The DASS-21^94,95^ is a 21-item self-report measure of psychological distress with three separate dimensions: depression (e.g., “I couldn’t seem to experience any positive feeling at all), anxiety (e.g., “I felt scared without any good reason”), and distress (e.g., “I found it hard to wind down”). Higher scores indicate greater levels of psychological distress. It was shown to have good construct validity and high reliability in non-clinical samples^96^.

#### Difficulties in emotion regulation scale—DERS

The DERS^97,98^ is a 32-item self-report measure of the modulation of emotional arousal, understanding, and acceptance of emotions, and the ability to act in desired ways regardless of emotional state. The six-factor structure of the DERS has been translated into six subscales: lack of emotional clarity (e.g., “I have difficulty making sense out of my feelings”); difficulty regulating behaviour when distressed (e.g., “When I’m upset, I become out of control”); difficulty engaging in goal-directed cognition and behaviour when distressed (e.g., “When I’m upset, I have difficulty getting work done”); unwillingness to accept certain emotional responses (e.g., “When I’m upset, I become angry at myself for feeling that way); and lack of access to strategies for feeling better when distressed (e.g., “When I’m upset, I believe there is nothing I can do to feel better”). DERS internal consistency is strong for all subscale except “awareness”^99^, thus we did not use the scoring from that subscale. Higher scores indicate greater levels of psychological distress.

#### Barratt impulsiveness scale-11—BIS-11

The BISS-11^62,100^ is a 30-item self-report measure of impulsiveness that yields six first-order factors which are attention, motor, self-control, cognitive complexity, perseverance, and cognitive instability impulsiveness. These factors form three second-order factors: attentional impulsiveness (e.g., “I don’t pay attention”); motor impulsiveness (e.g., “I act on impulse”); and non-planning impulsiveness (e.g., “I say things without thinking”). Higher scores indicate greater levels of impulsiveness. BIS-11 is widely used and has good reliability and validity^101^. While motor and non-planning impulsiveness can be considered independent factors, attentional impulsiveness may underly a general tendency towards impulsiveness^62^^.^

#### No go AX-continuous performance task—AX-CPT

The AX-CPT^102^ is a cognitive paradigm that distinguishes between proactive and reactive mechanisms of cognitive control. Participants respond to a probe based on a preceding cue. Each trial presents a cue letter followed, after a delay period, by a probe letter. Participants are asked to make a target response when they detect the “AX” sequence (an A cue followed by an X probe), and a non-target response to all other letter sequences (AY trials: an A cue followed by any probe other than X, BX trials: any cue other than A followed by an X probe, and BY trials: any cue other than A followed by any probe other than X). A critical feature of the design is that A cues and X probes are strongly associated due to a large proportion of AX trials, leading both to an increased target expectancy following an A cue, and to a prepotent target response tendency when presented with an X probe. This AX-CPT includes no-go stimuli as well. There are 4 different standard (go) trial types: AX, AY, BX, and BY. The proportions of trial types are set to ensure equal frequencies of A-cue and B-cue trials. No-go stimuli occur with low frequency and are indicated by digits (1–9) rather than letter probes (and equally follow A-cue and B-cue contexts). Participants are instructed to withhold responses in these trials. The presentation time of cues and probes is 500 ms. Probes are accompanied by a white rectangular border, presented 250 ms before probe onset. The cue-probe delay interval is 4 s, placing demand on goal (context) maintenance processes. The full condition includes 216 trials (72 AX, 18 AY, 18 BX, 72 BY, 18 A-No Go, 18 B-No Go). The hypothesis is that this manipulation would deter participants from making exclusive use of a proactive strategy, as preparing a response in advance would elicit more errors in the no-go trials. As a measure of proactive control, we use the Proactive Behavioural Index (PBI), which quantifies the relation between AY and BX reaction times in the AX-CPT^103^. Proactive control is related to reduced reaction times in BX trials because good performance in those trials implies that the B cue prepares enough to suppress the automatic response induced by probe X. Instead, increased reaction times on AY trials reflect a difficulty in suppressing the prepared response to cue A when a non-X probe appears. The PBI is calculated through the formula (AY − BX)/(AY + BX) and a positive score means that the participant shows a bias toward proactive vs. reactive control modality.

#### Cued go/no go task

The Cued Go/No Go Task^81^ is a behavioural paradigm that measures inhibition and cognitive control. Participants are required to perform an action based on certain stimuli (e.g., press a button—Go) and inhibit that action under a different set of stimuli (e.g., not press that same button—No-Go). Go trials with a high probability (‘Go cue’) and cues that predict No Go trials with a high probability (‘No Go cue’) are used. In general, commission errors are of particular interest in all Go/No Go tasks as a measure of cognitive control. In cued Go/No Go tasks, Go-cues are thought to generate a response tendency that speeds up correct responses in Go-trials but increases the likelihood of commission errors in subsequent No Go trials. As a result, the cued Go/No Go paradigm provides a sensitive measure of cognitive control. Participants are asked to press the spacebar when they see green (Go) but not blue (No Go) rectangles. The blue and green rectangles can be vertical or horizontal. The vertical rectangle has a high probability of being green (a Go trial) and the horizontal rectangle has a high probability of being blue (a No Go trial). Participants get information about the orientation of the rectangle (cue) shortly before the color of the rectangle is revealed. The full condition includes 250 trials (100 vertical cue-Go targets; 25 vertical cue-No Go targets; 100 horizontal cue-No Go targets; 25 horizontal cue-Go targets). The duration of the fixation cross lasts 800ms, the interstimulus interval between offset of fixation and onset of cue lasts 500ms, then participants have 1 s to respond, and the intertrial interval is 700ms. We use the number of No Go errors since those trials seem to be related to inhibition-related positivity^104^.

### Procedure

The online experiment included three phases conducted over three weeks. Participants were provided with instructions for accessing the experiments on Monday and had 7 days to complete each phase. They received questionnaires in the first week, the AX-CPT in the second week, and the go/no go task in the third week. The questionnaires were presented using Qualtrics^105^, an online survey tool, the AX-CPT using an ad-hoc website programmed in PHP built for this research, and the Go/No Go using Inquisit Player^106^, a psychological measurement software.

### Data analysis

#### Hypothesis 1

As a prerequisite for the mediation analyses, we used a bootstrapped Pearson correlation between the Proactive Behavioural Index of the AX-CPT, the DASS-21, and the DERS. In the same way, we compared the distribution of PBI scores with those of CAS-1 and PTQ. The rationale behind using two different self-report measures of perseverative thinking (CAS-1 and PTQ) and accounting for both psychological distress and difficulties in managing emotions (DASS-21 and DERS) relies on the multiparadigm point of view that characterizes our research. The CAS-1 was developed in the therapeutic field, for assessing and monitoring patients during treatment^8^ and involved also items about behaviours and beliefs associated with this construct. The PTQ was instead explicitly designed to assess the general construct of perseverative thinking, detached from any particular disorder-specific content^93^. Using these two self-report measures may permit us to understand our results in both clinical and research approaches. We used DASS-21 to account for a more general basic view of psychological distress, similar to negative affectivity^96^, the internal state that we feel when we have failed to achieve a goal or to avoid a threat, while the DERS reflects a multifaceted view of emotion dysregulation, considering different aspects of mental suffering^97^.

We employed a series of mediation analyses to assess the nature of the association between proactive control mode, perseverative thinking, and psychological distress. This regression-based path analysis tests hypotheses about how some antecedent variable X transmits its effect on a consequent variable Y through a third variable M. One pathway leads from X to Y without passing through M and is called the direct effect of X on Y. The second pathway from X to Y is the indirect effect of X on Y through M. A positive direct effect means that the case higher on X is estimated to be higher on Y when M is constant, while a positive indirect effect means that the case higher on X is estimated to be higher on M and the case higher in M is estimated to be higher in Y when X is constant^82^.

We performed four mediation analyses considering the effect of the PTQ in the PBI × DASS-21 association, the CAS-1 in the PBI × DASS-21 association, the PTQ in the PBI × DERS association, and the CAS-1 in the PBI × DERS association. As a control analysis, we tested a more reactive framework according to which psychological distress causes perseverative thinking which, in turn, influences the proactive control mode, considering the effect of the PTQ in the DASS-21 × PBI association, the CAS-1 in the DASS-21 × PBI association, the PTQ in the DERS × PBI association, and the CAS-1 in the DERS × PBI association. These control models are not meaningful, but in this way, we can test the specificity and the direction of our mediation analyses.

#### Hypothesis 2

As a prerequisite for the mediation analyses, we used a bootstrapped Pearson correlation between the number of errors in No Go trials, psychological distress (DASS-21), perseverative thinking (PTQ), and impulsiveness (BIS). We focused on these questionnaires because the CAS-1 contains items, that are useful in clinical settings, but that could be related to impulsiveness (e.g., “How often in the last week have you used alcohol/drugs in order to cope with your negative feelings or thoughts?”) and DERS contains an entire subscale related to difficulties in controlling impulsive behaviours when distressed. Thus, it would have been difficult to conceptually discern their relations with impulsiveness when interpreting the results.

A mediation analysis then tested if the relationship between inhibitory control (Go/No Go) and psychological distress (DASS-21) is mediated by perseverative thinking (PTQ) and/or impulsiveness (BIS). We further used a bootstrap pairwise comparison to directly test the size of indirect effects. Moreover, we implemented a similar mediation analysis to fractioning impulsivity effect in the three subcomponents proposed by Barratt^101^: attentional impulsiveness, motor impulsiveness, and non-planning impulsiveness.

Correlations were implemented using the software SPSS Statistics 25^107^ and mediation analyses were carried out through PROCESS macro model 4^82^. Analyses’ significance testing was performed using the bootstrap method (CI = 95%; bootstrap samples = 5000). The algorithm involves i. the random selection of observations from the data with replacement to create a resampled dataset of the same size as the original; ii. the computation of the analysis on the resampled dataset; iii. the repetition of the first two steps 5000 times to generate bootstrapped distribution; and iv. the computation of the confidence interval, a lower bound representing the 2.5th and an upper bound representing the 97.5th percentile of the bootstrapped distribution. If the confidence interval doesn’t contain zero, when the lower and the upper bounds have the same signs, it means that the effect is significantly different from zero with a confidence interval of 95%.

## Data Availability

The data that support the findings of this study are available from the corresponding author upon request.
